# Higher Consumption of Ultra-Processed Foods Is Associated with Lower Plant-Based Diet Quality in Australian Adults

**DOI:** 10.3390/nu17071244

**Published:** 2025-04-02

**Authors:** Natalia Tolstova, Priscila Machado, Laura E. Marchese, Katherine M. Livingstone

**Affiliations:** 1 School of Exercise and Nutrition Sciences, Deakin University, 221 Burwood Hwy, Burwood, VIC 3125, Australia; 2 Institute for Physical Activity and Nutrition (IPAN), School of Exercise and Nutrition Sciences, Deakin University, Geelong, VIC 3220, Australia; p.machado@deakin.edu.au (P.M.); l.marchese@deakin.edu.au (L.E.M.); k.livingstone@deakin.edu.au (K.M.L.)

**Keywords:** ultra-processed foods, the NOVA system, plant-based diets, plant-based diet quality, plant-based diet indices

## Abstract

**Background/Objectives**: Plant-based diets are associated with human and planetary health. However, the overall quality of these diets may depend on the proportion of ultra-processed foods (UPFs). This study investigates the association between UPF consumption and plant-based diet quality in Australian adults. **Methods**: Analysis was conducted on 9111 participants (aged ≥ 19 years) from the National Nutrition and Physical Activity Survey 2011–2012. Food items reported from a 24 h recall were classified using the NOVA system, and the contribution of UPFs to total energy intake was calculated. Plant-based diet quality was examined using the overall plant-based diet index (PDI), healthy PDI (hPDI), and unhealthy PDI (uPDI). Multiple linear regression models were used to examine the association between the UPF intake and PDI, hPDI, uPDI, and their score components. **Results**: The proportion of energy from UPFs was positively associated with the uPDI (β = 0.80; 95% CI 0.72, 0.89) and negatively associated with the PDI (β = −0.13; 95% CI: −0.22, −0.04) and the hPDI (β = −0.65; 95% CI: −0.73, −0.57). All score components of the PDI, hPDI and uPDI, except whole grains, legumes and fruit juices, significantly contributed to these associations. The sugars and syrups group contributed most to the positive association between UPFs and the uPDI (β = 6.47; 95% CI: 6.07, 6.87) and the negative association of UPFs and the hPDI (β = −6.47; 95% CI: −6.07, −6.87). **Conclusions**: Higher consumption of UPFs was associated with a lower plant-based diet quality. These findings have implications for the design of dietary interventions that encourage the consumption of minimally processed plant-based foods.

## 1. Introduction

Plant-based diets are characterized by higher intakes of fruits, vegetables, nuts, seeds, legumes, and whole grains and lower intakes of meat, poultry, seafood, and animal products [1,2,3]. Although research suggests that plant-based diets are more environmentally sustainable and associated with lower risk of non-communicable diseases and mortality than diets high in animal foods [4,5,6], research has evolved to recognize that not all plant foods are beneficial for human and planetary health [7]. Considerations for the quality of plant foods center predominantly on the level of processing and the addition of added sugars, salt, and saturated fat to foods and beverages [7]. To reflect these considerations, plant-based diet quality indices have been designed to positively score diets based on a low intake of processed plant foods, such as sugar-sweetened beverages (SSBs), and a high intake of whole plant foods, such as legumes [7]. A recent review of 35 plant-based diet quality indices demonstrated that the 20 indices differentiated between plant and animal foods based on their level of processing [7].

A rise in the adoption of plant-based diets among consumers globally may also signal a rise in ultra-processed foods (UPFs) [8,9], defined by NOVA as formulations of ingredients that result from a series of industrial processes [10]. When substituting animal foods for plant foods, consumers may increase their consumption of less-healthy plant foods, such as potato chips and refined grains, that are associated with adverse health outcomes [3,11]. The availability of plant-based alternatives to meat and milk products has also risen dramatically in supermarkets [8,12,13], and research suggests that most of these plant-based alternatives are high in saturated fat and added salt and are classified as UPFs [14,15,16,17]. Consequently, recent research suggests that plant-based diet quality declines as UPFs or likely-to-be UPFs consumption increases [18,19,20,21,22,23,24,25,26,27,28,29,30]. However, research on the contribution of UPFs to plant-based diets using validated indices and the NOVA methodology for food processing classification is limited [7,19,25,28]. Furthermore, given the many indices available to classify a plant-based diet and the range of minimally processed and ultra-processed plant foods available, the contribution of UPFs to plant-based diets is not well established. As more consumers globally are shifting towards more plant-based diets, understanding the contribution of UPFs to plant-based diets is important for informing public health plant-based dietary guidelines and interventions. Therefore, this project aimed to answer the question of whether there is a cross-sectional association between UPF consumption and plant-based diet quality in Australian adults. It is hypothesized that Australian adults with a higher intake of UPFs will have lower plant-based diet quality.

## 2. Materials and Methods

### 2.1. Study Design and Participants

This study is a secondary analysis of cross-sectional data collected during the National Nutrition and Physical Activity Survey (NNPAS) 2011–2012, part of the Australian Health Survey [31]. The NNPAS is a nationally representative survey that involved 12,153 (response rate: 77%) Australians ≥2 years old [31]. To select a nationally representative sample for the 2011–2012 NNPAS, a stratified multistage area sample of private dwellings in each state and territory of Australia was used [31]. The sampling included one adult (aged ≥ 18 years) and one child (aged ≥ 2–17 years) from each dwelling [31]. Trained interviewers collected data on socio-demographic, dietary, and lifestyle characteristics during face-to-face interviews with the selected adult member of the household [31]. The interview components of the NNPAS were conducted under the Census and Statistics Act (CSA) 1905.

**Figure 1:**
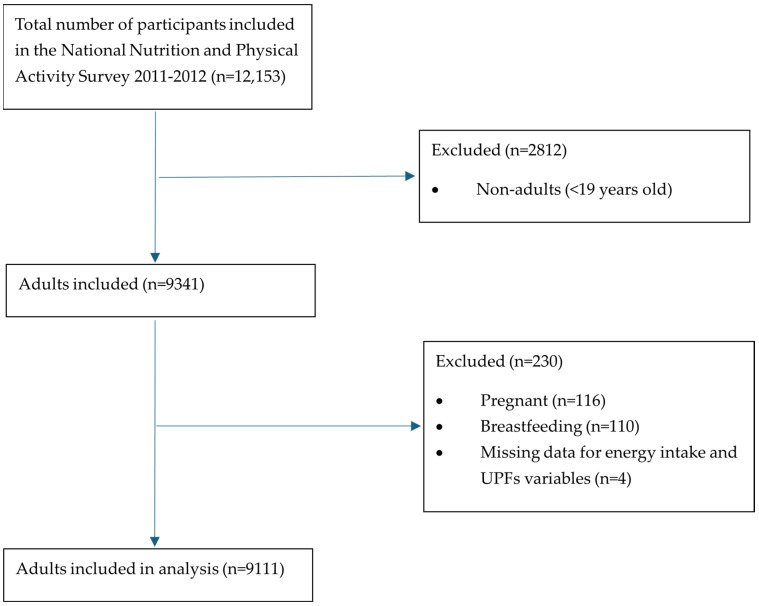
Flow diagram of subjects from the 2011–2012 National Nutrition and Physical Activity Survey included in the analysis.

In this study, participants were excluded from the analysis if they were less than 19 years of age (*n* = 2812), pregnant (*n* = 116), breastfeeding (*n* = 110), or missing data for any variables required for analysis (*n* = 4 missing data for UPF intake). The cut-off age of 19 years for exclusion from the adult population was chosen to be consistent with age groups used in the reporting by the Australian Bureau of Statistics [32], the Australian Institute for Health and Welfare [33], and the Australian Nutrient Reference Values [34]. A total of 9111 subjects were included in this study (Figure 1). The study results are reported using the Strengthening the Reporting of Observational Studies in Epidemiology—Nutritional Epidemiology (STROBE-nut) reporting guidelines (Supplementary Table S1) [35].

### 2.2. Dietary Assessment

Dietary intake data were collected via two 24 h recall dietary assessments, using an adapted version of the US Automated Multiple Pass Method [31]. The first recall was conducted during the face-to-face interview (*n*  =  12,153 participants), and the second 24 h recall was repeated via telephone (*n*  =  7735 participants, response rate: 63.6%) at least eight days after the initial recall, targeting different days of the week [31]. The Australian Food and Nutrient Database (AUSNUT 2011–2013) was used to estimate energy intakes [36]. Foods were assigned an eight-digit food code and classified into five major, sub-major, and minor food groups [31]. Only data from the first dietary recall was used in this study to avoid selection bias, as the second recall had a lower response rate (63.6%) [31].

### 2.3. Plant-Based Diet Quality

Overall plant-based diet index (PDI), healthy plant-based diet index (hPDI), and unhealthy plant-based diet index (uPDI), originally created by Satija et al. [26], were used to assess the plant-based diet quality. An Australian plant-based diet database created by Stanford et al. was used to supplement the NNPAS data as it was compatible with the codes of foods and beverages used [37], and has been used to estimate PDI, hPDI, and uPDI in the Australian population [38]. This database categorizes all plant and animal content of single- and multi-ingredient foods, beverages, and mixed dishes into 23 plant and animal food groups for each participant [37]. However, in the present study, we combined low-, medium-, and high-fat dairy food groups into one dairy group, and red meat and poultry with different fat content into one meat group. This was performed to classify the participants’ diets into 18 groups, as initially proposed by Satija et al. [26]. Whole grains, fruits, vegetables, nuts and seeds, legumes, unsaturated plant oils/spreads, and tea and coffee were classified as healthy plant food groups. In contrast, refined grains, fruit juices, saturated plant fats, sugars and syrups and miscellaneous plant products were classified as unhealthy plant foods. Animal food groups included animal fats, dairy, eggs, fish and seafood, red meat and poultry and miscellaneous animal-based food items [26].

The total number of servings per person for each of the 18 food groups was divided into population-specific quintiles, and each quintile received a score from 1 to 5 [26]. When calculating the overall PDI, participants received a score of five for each plant food group they consumed within the highest quintile of consumption (regardless of whether it is healthy or not), a score of four for each plant food group consumed within the second-highest quintile, and so on. In contrast, each animal food group was reverse-scored with a score of 1 and 5 applied to the highest and lowest consumption quintiles, respectively. For hPDI, positive scores were given to healthy plant food groups and reverse scores to less healthy plant food groups and animal food groups. Finally, for uPDI, positive scores were given to less healthy plant food groups and reverse scores to healthy plant food groups and animal food groups. To calculate PDI, hPDI and uPDI, scores for all components were summed for each participant, with a possible range between 18 and 90. More details on food group classification and PDI, hPDI and uPDI calculation are presented in Supplementary Table S2 and Supplementary Figure S1 and described elsewhere [26].

### 2.4. UPFs Classification

All food and beverage items from AUSNUT 2011–2013 had been previously classified according to the NOVA food classification system [39]. In short, all single food items and the individual ingredients from handmade recipes were classified according to the four NOVA system groups: (1) unprocessed or minimally processed foods (examples: fruits, nuts, cereals and eggs); (2) processed culinary ingredients (examples: oils and table sugar); (3) processed foods (examples: cheese, canned fruit and vegetables, and processed bread); (4) UPFs (examples: frozen and shelf-stable ready meals, soft drinks, breakfast ‘cereals’, and confectionary) [39]. Food items were ultimately classified as UPFs if they contained ingredients derived from foods but of non-culinary use (e.g., protein isolate, inverted sugar, hydrogenated oil) or classes of additives with cosmetic functions (e.g., colors, flavors, emulsifiers, artificial sweeteners). Information present in the list of ingredients of food items sold in the Australian supermarket’s websites were used for classification, and most of the plant-based alternatives available were classified as UPFs. The recipes for all mixed dishes, identified as handmade, were broken down using the AUSNUT 2011–2013 Food Recipe File allowing the classification of ingredients into one of four NOVA food groups. More details on how handmade mixed-dishes were disaggregated into underlying ingredients and the application of the NOVA system in the AUSNUT 2011–2013 are described by Machado et al. [39]. The contribution of UPFs to the total energy intake (% energy/day) was estimated and used in the analysis.

### 2.5. Socio-Demographic Characteristics

Australian Bureau of Statistics-trained interviewers collected socio-demographic data for all participants via face-to-face interviews [31]. A range of descriptive demographic characteristics, including age, sex, education, area-level disadvantage, rurality, and country of birth, was captured. Country of birth was categorized as Australia, main English-speaking country (Canada, Republic of Ireland, New Zealand, South Africa, UK and USA), or other [31]. Area-level disadvantage was calculated using the ABS Index of Relative Socio-economic Disadvantage (SEIFA 2011—National) and divided into quintiles, where the lowest 20% represented the greatest disadvantaged group and the highest 20% represented the most advantaged group [31]. Level of education was divided into tertiles—low (incomplete high school or less), medium (complete high school or incomplete high school and/or certificate/diploma) and high (tertiary qualification). Rurality was divided into three categories—major cities of Australia, inner regional Australia, and other (outer regional Australia, remote Australia, and very remote Australia) [31].

### 2.6. Data Analysis

Descriptive statistics (mean and standard error for continuous variables and % for categorical variables) were used to describe demographic characteristics, energy intake, and proportion of total energy from UPFs according to quintiles of the PDI, hPDI, and uPDI. Crude (unadjusted) and multivariate (adjusted) *p*-values were calculated for linear trends across groups by applying linear regression modeling for continuous variables and Pearson’s χ^2^ for categorical variables.

Crude and multiple linear regression analyses were used to examine the association between the proportion of the total energy intake from UPFs (continuous dependent variable) and each index (PDI, hPDI and uPDI; continuous independent variables). Model 1 was adjusted for covariates identified using a directed acyclic graph (Supplementary Figure S2), and they included age (continuous), sex (binary), education level (categorical), country of birth (categorical), rurality (categorical), and area-level disadvantage (categorical). Energy misreporters were not excluded from the analysis as it could lead to selection bias due to differences in characteristics of plausible and non-plausible energy reporters [40]. Instead, model 2 was adjusted for energy misreporting (continuous), which was defined as an energy intake to basal metabolic rate ratio [40].

To assess the contribution of plant-based diet quality components to UPF consumption, studied participants were divided according to quintiles of dietary energy share of UPFs. The first and fifth quintiles represented consumers with the lowest and highest consumption of UPFs (% energy/day), respectively. Then, the contribution of score components for PDI, hPDI, and uPDI was calculated according to quintiles of UPF consumption. Also, associations between the proportion of energy from UPFs (continuous) and score components for PDI, hPDI, and uPDI (continuous) were examined using multiple linear regression analyses, adjusted for age, sex, education level, country of birth, rurality, area-level disadvantage, and energy misreporting.

Interaction terms were added to the models to evaluate moderation of associations between the proportion of total energy intake from UPFs and each diet quality index (PDI, hPDI and uPDI) by age, sex and level of education.

To extrapolate the results to the general Australian adult population, sampling and replicate weights were applied to all statistical analyses to account for selection bias and the effect of the sampling method used in the NNPAS [31]. Statistical significance was set at *p* < 0.05. Statistical software Stata (v18, Stata Corp, College Station, TX, USA) was used for data analyses.

## 3. Results

### 3.1. Participants Characteristics

A total of 9111 (49.4% female, mean (SE) age 49.4 (17.3) years) participants from 2011–2012 NNPAS were included in the analysis. The mean (SE) energy intake of the participants was 2001 (12.2) kcal per day, and the mean (SE) contribution of UPFs to total daily energy intake was 39.1% (0.3). In the total sample, the PDI ranged from 28 to 78 (mean (SE): 52.7 (0.1)), the hPDI ranged from 30 to 80 (mean (SE): 54.8 (0.1)), and the uPDI ranged from 32 to 81 (mean (SE): 56 (0.1)) (possible range 18 to 90).

Characteristics of the studied population according to quintiles of PDI, hPDI, and uPDI are shown in Table 1. Participants within the highest quintile of PDI (ranging from 59 to 78) had a higher energy intake (*p* trend < 0.001) and a lower dietary contribution of UPFs (*p* trend < 0.001). The higher the quintile of PDI, the older the participants (*p* trend = 0.002) and the higher the prevalence of participants who had higher education (*p* trend < 0.001), were born in Australia (*p* trend = 0.003), and lived in major cities of Australia (*p* trend = 0.016). No statistically significant differences were observed in sex and area-level disadvantage characteristics among participants across quintiles of PDI.

Participants within the highest quintile of hPDI (ranging from 62 to 80) had lower energy intake (*p* trend < 0.001) and lower dietary contribution of UPFs (*p* trend < 0.001) and were older (*p* trend < 0.001). As the quintile of hPDI increases, the prevalence of female subjects rises from 40.8% of females in the lowest quintile to 58.6% of females in the highest quintile of hPDI (*p* trend < 0.001). No statistically significant differences were observed in the country of birth, rurality, or area-level disadvantage characteristics among participants across quintiles of hPDI.

Participants within the highest quintile of uPDI (ranging from 63 to 81) had lower energy intake (*p* trend < 0.001), and a higher dietary contribution of UPFs (*p* trend < 0.001), were younger (*p* trend < 0.001), had lower levels of education (*p* trend < 0.001), were more likely to be not born in a main English-speaking country (*p* trend < 0.001), and experienced the greatest area-level disadvantage (*p* trend < 0.001). No statistically significant differences were observed in the sex and rurality characteristics among participants across quintiles of uPDI.

### 3.2. UPFs and PDI, hPDI and uPDI

#### 3.2.1. Intake of Plant-Based Diet Quality Components and UPFs

**Figure 2:**
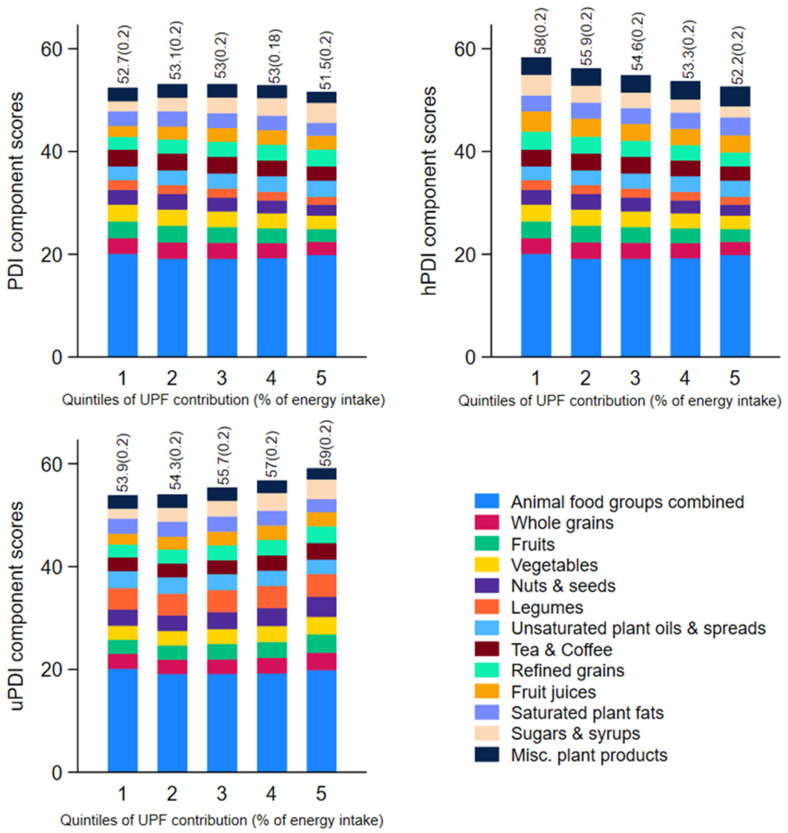
Plant-based diet indices component scores across quintiles of the percentage of energy from ultra-processed foods in Australian adults from the 2011–2012 NNPAS (*n* = 9111). Mean (Range) contribution from UPFs: Q1 = 11.2 (0–19.3), Q2 = 25.5 (19.3–31.4), Q3= 37.0 (31.4–42.9), Q4 = 50.2 (42.9–58.6), Q5 = 71.6 (58.6–100). Values above columns show the mean (SE) total diet quality score, which theoretically could range between 0 and 90, with a higher score indicating higher adherence to respective diets. Some components scored inversely (refer to Supplementary Figure S1 and Supplementary Table S2).

The mean (SE) scores for PDI, uPDI, and hPDI, as well as the component scores for each index, according to quintiles of energy from UPFs, are shown in Figure 2. Component scores are also presented in Supplementary Table S3. The greatest difference in the mean (SE) component scores from the first to the fifth quintile of UPFs was for the sugars and syrups group. For PDI and uPDI this ranged from 2.0 (0.03) to 3.8 (0.04), whereas for hPDI this ranged from 4.0 (0.04) to 2.2 (0.04). The second greatest difference across quintiles of UPFs was the nuts and seeds food group, where the mean (SE) component score for PDI and hPDI ranged from 3.0 (0.05) to 2.1 (0.05) and for uPDI from 3.1 (0.06) to 3.9 (0.05). The third greatest difference across quintiles of UPFs was observed in refined grains: mean (SE) component score for PDI and uPDI ranged from 2.6 (0.05) to 3.4 (0.04) and for hPDI this ranged from 3.4 (0.05) to 2.6 (0.04), followed by fruits and vegetables group (Supplementary Table S3).

#### 3.2.2. Association Between UPFs and PDI, hPDI, and uPDI

Table 2 presents the results from the crude and multivariable linear regression analyses assessing the associations between the proportion of energy from UPFs and PDI, hPDI, and uPDI in Australian adults. The crude, multivariable Model 1 (adjusted for sociodemographic variables) and multivariable Model 2 (adjusted for Model 1 and energy misreporting) showed that the proportion of energy from UPFs was significantly positively associated with uPDI (Model 2: β = 0.80, 95% CI: 0.72, 0.89, *p* < 0.001) and significantly negatively associated with PDI (Model 2: β = −0.13, 95% CI: −0.22, −0.04, *p* = 0.005) and hPDI (Model 2: β = −0.65, 95% CI: −0.73, −0.57, *p* < 0.001).

Supplementary Table S4 presents the results from the moderation analyses and subgroup analyses according to age, sex and education. Evidence of an interaction was observed for UPFs and PDI by age and education and for UPFs and uPDI by sex and education.

#### 3.2.3. Association Between UPFs and Component Scores

Crude and multivariable models showed that the proportion of energy from UPF was associated with all component scores of PDI, hPDI and uPDI except for wholegrains, legumes and fruit juices (Table 3). From healthy food groups, the vegetables contributed most to the negative association of UPFs with PDI and hPDI (β = −2.32; 95% CI: −2.70, −1.94), and the positive association of UPFs with uPDI (β = 2.32; 95% CI: 1.94, 2.70), followed by fruits (β = −1.56; 95% CI: −1.93, −1.21), and nuts and seeds (β = −1.55; 95% CI: −1.86, −1.24) groups (Table 3). On the opposite, the unsaturated plant oils and spreads group was significantly positively associated with UPFs and attenuated the negative association of UPFs with PDI and hPDI (β = 1.79; 95% CI: 1.38, 2.19) and positive association of UPFs with uPDI (β = −1.79; 95% CI: −2.19, −1.38).

From unhealthy food groups, the sugars and syrups contributed the most to the positive association of UPFs with uPDI (β = 6.23; 95% CI: 5.84, 6.63), to the negative association of UPFs with hPDI (β = −6.23; 95% CI: −6.63, −5.84,), as well as attenuated the magnitude of negative association of UPFs and PDI (β = 6.23; 95% CI: 5.84, 6.63), followed by refined grains (β = 1.58; 95% CI: 1.17, 1.99). Conversely, the saturated plant fats group was significantly negatively associated with UPFs and attenuated the positive association of UPFs with uPDI (β = −1.58; 95% CI: −1.93, −1.23).

## 4. Discussion

This cross-sectional analysis of a nationally representative sample of Australian adults found that the proportion of energy from UPFs was positively associated with uPDI and negatively associated with PDI and hPDI, confirming the hypothesis that Australian adults with higher intake of UPFs have a lower plant-based diet quality. Findings from this study also identified that the sugars and syrups food group contributed most to the positive association of UPFs with PDI and uPDI and to the negative association of UPFs with hPDI, highlighting the importance of limiting the intake of less healthy plant-based items, such as SSBs. This nationally representative study is among the first to show associations between UPFs and plant-based diets using validated plant-based diet quality indices and the NOVA classification system, providing population-level insights into two topical dietary components. These findings have implications for the design of dietary interventions and healthy eating policies that encourage the consumption of minimally processed plant-based foods.

The findings from the present study are aligned with previous research on UPFs or likely-to-be UPFs and plant-based diet quality assessed by PDIs [19,21,25,26,27]. For example, two cross-sectional studies conducted in France using data from the NutriNet-Santé cohort [19] and from a French national survey (INCA3, 2014–2015) [25], showed that lower adherence to hPDI was associated with higher UPF contribution to participants’ diets. The same association between UPFs and hPDI was reported in a cross-sectional analysis of 121,300 adults in the UK Biobank [28]. Similarly, three US studies reported that higher adherence to uPDI was associated with a higher intake of processed foods (likely-to-be UPFs), such as SSBs, refined grains, sweets, and desserts, although these studies did not use the NOVA classification system [21,26,27]. Furthermore, several studies that assess diet quality using other indices (the Dietary Obesity-Prevention score, the American Cancer Society diet score, and a priori diet quality score) that reverse-scored processed foods such as refined grains, sweets and desserts, salty snacks, and SSBs reported a negative association between the diet quality and these foods, which is consistent with the results of the current study [20,22,23]. A recent nationally representative study conducted on 46,164 Brazilians over 10 years of age showed an inverse association between the consumption of UPFs (using the NOVA system) and the Planetary Health Diet Index [41]. Differences in the classifications of foods between these studies highlight the importance of applying a standardized scoring system, such as the NOVA system, when categorizing foods according to the level of industrial food processing, enabling the assessment of underestimated food groups and comparisons among different studies [42].

The current study showed a significant negative association between UPF contribution and adherence to overall PDI in the Australian adult population; however, the magnitude of this association is smaller than that between UPFs and hPDI. The same negative trend between UPF contribution and PDI was found in the analysis of data from a French national survey [25]. Interestingly, the study conducted in the UK Biobank found a significant positive association between PDI and UPF consumption [28]. As PDI reflects adherence to all plant foods and does not differentiate healthy and unhealthy plant foods, there might be several possible explanations for discrepancies within these findings. Firstly, it may reflect the differences in UPF intake between countries, where UPF contribution from both plant and animal-based products to total energy intake is estimated to be lower in France (17–33%) than in Australia (42%) and the UK (nearly 60%) [43]. Secondly, the participants of the UK study are from a large-scale volunteer databank, which may be biased towards white ethnicity and highly educated older participants with higher income who have more purchasing power for plant-based alternatives than younger participants with lower income [44].

Even though a negative association between UPF intake and overall PDI was found in the present study, the magnitude of this association is much smaller than between UPF intake and hPDI. Considering that the number of plant-based meat substitute products available in Australian, US and European supermarkets has increased and is anticipated to grow [9,12,45,46], and UPFs sales are rising globally [47], the results from the recent data could show a positive trend between UPFs and PDI, similar to previous research [28]. For example, studies conducted among French participants from the NutriNet-Santé cohort (*n* = 21,212) and German adults (*n* = 814) that investigated the contribution of UPFs to different plant-based diet types found that vegans consumed the most plant-based meat and dairy substitutes followed by vegetarians [19,24]. Such findings were expected as these food products are designed to substitute those of animal origin and are marketed to consumers following PBDs [48]. Moreover, the result of the French study found that vegetarians and vegans had a higher UPF intake by consuming higher amounts of salty snacks and biscuits [19]. Also, recently published analyses of the UK Biobank found significantly higher UPF consumption in vegetarians compared to meat-eaters [29]. Conversely, the German study reported that convenience products, fast foods, snacks, and ultra-processed beverages contributed significantly more to meat-rich diets [24]. Despite consuming more processed foods from different subgroups, the diet quality that was assessed using hPDI [19] and HEI-flex [18] increased with higher avoidance of animal-based foods in the French and German studies, respectively. This highlights the relevance of studies that evaluate the contribution of different processed foods, including plant-based meat and dairy alternatives, to plant-based diets.

The results of the current study show that among all score components of PDIs, the sugars and syrups group, followed by the refined grains group, contributed most to the positive association between UPFs and uPDI. Furthermore, despite the negative association between PDI and UPFs, the contribution of the sugars and syrups group and the refined grains increases with higher PDI scores. So, participants with higher levels of adherence to overall and unhealthy PBDs have a higher consumption of sugars and syrups, contributing significantly to their UPF intake. These findings align with a US study conducted in a nationally representative sample of adults, where the intake of SSBs and refined grains increased with higher adherence to PDI and uPDI [21]. Interestingly, in another study of three US cohorts, intake of refined grains increased with higher PDI, but no differences were reported for SSBs across deciles of PDI [26]. However, no statistical analyses were performed to check the significance of such trends reported in this study, and participants were health professionals who could be more aware of the negative health impact of SSBs consumption [26].

Among healthy score components, a lack of vegetables, followed by a lack of fruits and nuts and seeds, contributed most to the positive association between UPFs and uPDI. These findings were expected as it is consistently reported in the literature that UPFs tend to displace consumption of whole foods as UPF intake increases progressively [39,49]. However, higher consumption of both UPFs and unprocessed foods, such as fruits, vegetables, legumes, nuts and others, was reported among vegans compared to meat-eaters in the French NutriNet-Santé cohort study [19]. This could be explained by vegans consuming a smaller dietary share of processed foods (classified as group 3 by the NOVA) compared to meat eaters, although this was not reported by the authors [19].

There are several known mechanisms through which UPFs may impact diet quality, such as high content of saturated fat, added sugar, energy density, and salt, and low fiber and micronutrient content [11]. Furthermore, UPFs replace whole nutritious foods in diets, such as fruits, vegetables, nuts, and legumes [50]. Such nutrient-poor dietary profiles are known to impact the risk of developing chronic diseases via physiological pathways, including elevated inflammation and oxidative stress, disrupting gut microbiota and impairing metabolic regulation [50,51]. However, the physiological pathways through which UPFs impact diet quality go beyond their nutrient composition and energy density, and include the physical and chemical properties related to industrial processing methods [50,51].

This study has implications for policy, practice, and individual dietary behaviors. Delivering healthy foods from sustainable food systems is essential in realizing multiple United Nations’ Sustainable Development Goals, with the Food and Agriculture Organisation of the United Nations and the World Health Organization recommending restricting highly processed foods and drinks and prioritizing plant- versus animal-based diets within their guiding principles for sustainable healthy diets [52]. Plant-based healthy eating policies should encourage intake of whole plant foods such as legumes, fruits, and vegetables, and limit intake of UPFs. Furthermore, as the demand and availability of plant-based substitutes for animal products grows, food composition databases will require regular updates to reflect the plant-based alternatives in the food supply [53] and regular monitoring and surveillance of the consumption of plant-based alternatives in population diets are required. A similar approach should be implemented at an individual level for dietary behaviors. For example, health professionals should advise that the composition of plant-based foods and overall diet quality are important considerations when adopting a plant-based diet. In addition, information on the quality of plant-based products should be available to consumers so that they can make informed decisions when purchasing these foods.

The findings from this study have implications for future research. In particular, this analysis should be repeated using upcoming national dietary data releases to evaluate UPF contribution to plant-based diets in the current food supply. Considering the exponentially growing market of industrial plant-based meat and dairy alternatives, further research should assess the contribution of these specific groups of products to plant-based diets. Future studies should investigate longitudinal relationships between the UPF contribution to plant-based diets and health outcomes, for example, if the proportion of UPFs mediates the effect of plant-based diets on diet-related chronic diseases. Research in this field would also benefit from evidence of the health impacts of plant-based foods according to the degree of processing and nutrient content. For example, a large-scale cohort study found a direct association between plant-sourced UPFs and cardiovascular disease risk, and the inverse between plant-sourced non-UPFs and cardiovascular disease risk [54]. In contrast, Cordova et al. reported that ultra-processed plant-based alternatives were not associated with a risk of cancer-cardiometabolic multimorbidity [55], and evidence for the role of nutrient content is lacking. Therefore, further research is necessary to investigate this point. Furthermore, the NOVA system does not account for the nutrient content when classifying foods, despite evidence showing that higher UPF consumption is associated with poorer nutritional quality of diets [56]. Future research investigating the health impacts of PBDs based on the degree of processing and nutrient content would be valuable. Lastly, given the rapid rise in obesity in children and adolescents [57], further research should repeat this analysis using data on children and adolescents to understand these findings within the context of other age groups.

The current study has many strengths. The use of the nationally representative data offers some insights into the generalizability of these findings to the Australian population. The NOVA system to classify food by degree of processing was applied in the current study to identify UPFs. This system has been utilized in many studies to assess the quality of diets and health outcomes, and is widely recognized among researchers worldwide [42]. Another strength is the use of PDI metrics that allow the evaluation of plant food quality. In particular, hPDI and uPDI have been extensively utilized in assessing diet quality and its relation to chronic disease risk [21,26,27,28]. Finally, using the recently developed Australian plant-based database that segregates mixed dishes into individual foods contributed to a greater accuracy of PDI calculations [37].

There are several limitations in this study. Firstly, only the first day of the 24 h recall was used. A single dietary assessment may not adequately represent the long-term dietary intake; however, it is an appropriate tool for measuring dietary intake on a population level, where a large sample size attenuates the variation in intakes during the week [58]. Secondly, the proportion of plant-based meat and dairy substitutes in UPFs and their contribution to plant-based diets was not assessed. However, considering the limited availability of such alternatives at that time point, it is unlikely to influence our results. Thirdly, 2011–2012 NNPAS data were collected over a decade ago and may not reflect the current dietary intake or food supply of the Australian population. Finally, despite the use of a systematic approach to identify UPFs [39], the dietary assessment tool was not designed to measure UPFs specifically. Thus, some misclassification of items cannot be eliminated.

## 5. Conclusions

This cross-sectional study of a nationally representative sample of Australian adults showed that UPF consumption was inversely associated with plant-based diet quality. A lower intake of UPFs was associated with a higher healthy plant-based diet (assessed using hPDI), while a higher intake of UPFs was associated with higher unhealthy plant-based diet (assessed using uPDI). A higher adherence to an overall plant-based diet (assessed using PDI) was associated with a lower consumption of UPFs. This study also found that among all score components for PDIs, the sugars and syrups group, followed by the refined grains group, contributed most to the positive and negative association of UPFs with uPDI and hPDI, respectively. The findings outline the importance of processing when examining a plant-based diet, which has implications for the design of policies and programs to support healthy and sustainable plant-based diets. Future research should examine the longitudinal relationships between the contribution of UPFs to plant-based diets and health outcomes.

## Figures and Tables

**Table 1:** Characteristics of study participants (*n* = 9111) according to quintiles of three plant-based diet quality indices: PDI, hPDI, and Updi ^a^.

Characteristic	Plant-Based Diet Quality Index					
	Q1	Q2	Q3	Q4	Q5	*p* Trend
PDI						
*n* ^ (% weight adjusted)	1977 (21.3)	1984 (21.9)	1692 (18.5)	1806 (19.7)	1652 (18.6)	
PDI score	44 (0.08)	49.6 (0.03)	53 (0.03)	56.4 (0.04)	62.1 (0.1)	<0.001
Energy intake (kcal/d)	1744 (24.8)	1861 (25.2)	1953 (26.5)	2178 (27.8)	2320 (28.0)	<0.001
UPF intake (%E) ^^	41.0 (0.7)	40.3 (0.7)	37.7 (0.7)	39.5 (0.7)	36.3 (0.6)	<0.001
Age, y	45.2 (0.5)	46.3 (0.5)	47.3 (0.6)	46.6 (0.6)	48.3 (0.6)	0.002
Female, %	51.3	49.2	51.3	49.2	45.7	0.095
Education ^&^						
Low, %	28.2	27.4	29.3	22.3	21.5	<0.001
Medium, %	50.2	50.0	46.1	51.3	47.7	
High, %	21.6	22.6	24.6	26.4	30.9	
Country of birth ^<^						
Australia, %	73.3	66.2	69.5	68.5	66.4	0.003
English-speaking, %	11.6	13.1	10.9	10.8	11.4	
Other, %	15.1	20.7	19.5	20.7	22.2	
Rurality						
Major city, %	68.9	74.3	69.8	71.1	73.7	0.016
Inner regional, %	19.8	17.3	19.7	20.1	18.9	
Other, %	11.4	8.4	10.5	8.8	7.4	
Area-level disadvantage ^$^						
First quintile *, %	21.2	17.1	17.4	19.1	15.6	0.193
Third quintile, %	20.6	20.0	20.7	21.2	21.0	
Fifth quintile **, %	19.6	23.3	22.2	22.2	24.8	
hPDI						
*n* ^ (% weight adjusted)	2078 (24.2)	1773 (20.1)	1860 (20.3)	1599 (16.8)	1801 (18.5)	
hPDI score	45 (0.1)	52 (0.04)	56 (0.03)	59 (0.04)	66 (0.12)	<0.001
Energy intake (kcal/d)	2397 (27)	2063 (27)	1922 (26)	1773 (24)	1709 (24)	<0.001
UPF intake (%E) ^^	46.1 (0.6)	41.6 (0.7)	39.0 (0.7)	34.9 (0.7)	30.8 (0.7)	<0.001
Age, y	40.7 (0.5)	44.8 (0.5)	48.2 (0.6)	49.7 (0.6)	52.1 (0.5)	<0.001
Female, %	40.8	46.6	49.3	55.0	58.6	<0.001
Education ^&^						
Low, %	23.2	24.4	27.0	27.5	27.9	<0.001
Medium, %	51.5	49.9	51.4	48.5	43.3	
High, %	25.3	25.7	21.6	24.1	28.8	
Country of birth ^<^						
Australia, %	70.6	71.3	69.7	71.9	74.7	0.190
English-speaking, %	20.0	19.4	20.1	18.5	17.2	
Other, %	9.4	9.2	10.2	9.5	8.1	
Rurality						
Major city, %	70.6	71.3	69.7	71.9	74.7	0.425
Inner regional, %	20.0	19.4	20.1	18.5	17.2	
Other, %	9.4	9.2	10.2	9.5	8.1	
Area-level disadvantage ^$^						
First quintile *, %	19.3	17.7	17.7	17.8	17.7	0.761
Third quintile, %	21.0	22.0	20.4	20.5	19.3	
Fifth quintile **, %	21.8	21.4	20.9	24.5	23.8	
uPDI						
*n* ^ (% weight adjusted)	2057(21.7)	1859 (20.3)	1992 (21.1)	1663 (18.4)	1540 (18.5)	
uPDI score	46.5 (0.09)	52.6 (0.04)	56.5 (0.03)	60.4 (0.04)	66.2 (0.11)	<0.001
Energy intake (kcal/d)	2320 (25)	2109 (26)	1979 (25.4)	1813 (27)	1719 (28)	<0.001
UPF intake (%E) ^^	29.8 (0.5)	36.9 (0.6)	40.0 (0.6)	43.0 (0.8)	47.2 (0.8)	<0.001
Age, y	50.6 (0.5)	48.5 (0.5)	47.4 (0.5)	45.4 (0.6)	40.7 (0.6)	<0.001
Female, %	51.1	47.0	49.6	49.3	49.8	0.447
Education ^&^						
Low, %	21.5	24.4	25.6	29.9	28.5	<0.001
Medium, %	45.0	49.5	49.8	48.1	53.7	
High, %	33.4	26.1	24.6	21.9	17.8	
Country of birth ^<^						
Australia, %	69.7	67.9	68.0	69.4	69.2	<0.001
English-speaking, %	14.2	13.3	12.2	10.4	7.2	
Other, %	16.1	18.8	19.8	20.2	23.6	
Rurality						
Major city, %	69.8	70.7	72.9	73.1	71.5	0.076
Inner regional, %	21.0	19.7	16.9	17.2	20.8	
Other, %	9.2	9.6	10.2	9.7	7.7	
Area-level disadvantage ^$^						
First quintile *, %	11.7	17.7	18.9	20.2	23.1	<0.001
Third quintile, %	21.1	21.0	22.4	19.0	19.6	
Fifth quintile **, %	26.8	24.5	22.1	21.1	16.2	

Data are mean (SE) for continuous variables and percentage for categorical variables. Abbreviations: Q, quintile; PDI, plant-based diet index; hPDI, healthful plant-based diet index; uPDI, unhealthful plant-based diet index; PDI score range (Q1: 28–47, Q2: 48–51, Q3: 52–54, Q4: 55–58, Q5: 59–78), hPDI score range (Q1: 30–49, Q2: 50–53, Q3: 54–57, Q4: 58–61, Q5: 62–80) and uPDI score range (Q1: 32–50, Q2: 51–54, Q3: 55–58, Q4: 59–62, Q5: 63–81) with possible ranges between 18 and 90, where a higher score indicating better adherence to the respective diet. ^a^ Weighted analyses were conducted to apply the results to the Australian population at the time of the survey; ^ Unweighted sample number. ^^ % of total daily energy intake. ^&^ Low (incomplete high school or less), medium (completed high school or incomplete high school and/or certificate/diploma) and high (tertiary qualification). ^<^ Main English-speaking countries including Canada, Ireland, NZ, South Africa, UK, and US. ^$^ Calculated using the 2011 Index of Relative Socio-economic Disadvantage. * greater disadvantage, ** greater advantage.

**Table 2:** Associations between the proportion of energy intake from UPFs and three diet quality indices (PDI, hPDI and uPDI) in Australian adults from NNPAS 2011–2012 (*n* = 9111).

UPF, % Energy Intake	PDI		hPDI		uPDI	
	β (95% CI)	*p*	β (95% CI)	*p*	β (95% CI)	*p*
Crude	−0.23 (−0.32, −0.13)	<0.001	−0.75 (−0.83, −0.67)	<0.001	0.87 (0.79, 0.96)	<0.001
Model 1	−0.13 (−0.22, −0.04)	0.006	−0.65 (−0.73, −0.57)	<0.001	0.80 (0.71, 0.88)	<0.001
Model 2	−0.13 (−0.22, −0.04)	0.005	−0.65 (−0.73, −0.57)	<0.001	0.80 (0.72, 0.89)	<0.001

Abbreviations: PDI, plant-based diet index hPDI, healthful plant-based diet index, uPDI, unhealthful plant-based diet index; CI, Confidence Interval; Values are regression coefficients (95% CIs) estimated with multiple linear regressions. Model 1: Linear regression analyses adjusted for sociodemographic variables (sex, age, education, country of birth, rurality, and area-level disadvantage). Model 2: Adjusted for Model 1 + energy misreporting (total energy intake to basal metabolic rate ratio).

**Table 3:** Associations between the proportion of energy from UPFs and component scores for PDI, hPDI and uPDI in Australian adults from the NNPAS 2011–2012 (*n* = 9111).

	PDI	hPDI	uPDI	
Food Groups	β (95% CI)			*p* Value
Healthy plant food groups				
Whole grains	0.09 (−0.30, 0.47)		−0.09 (0.47, −0.30)	0.652
Fruits	−1.56 (−1.93, −1.21)		1.56 (1.21, 1.93)	<0.001
Vegetables	−2.32 (−2.70, −1.94)		2.32 (1.94, 2.70)	<0.001
Nuts and seeds	−1.55 (−1.86, −1.24)		1.55 (1.24, 1.86)	<0.001
Legumes	−0.09 (−0.39, 0.21)		0.09 (−0.21, 0.39)	0.560
Unsaturated plant oils and spreads	1.79 (1.38, 2.19)		−1.79 (−2.19, −1.38)	<0.001
Tea and Coffee	−1.51 (−1.88, −1.14)		1.51 (1.14, 1.88)	<0.001
Unhealthy plant food groups				
Refined grains	1.58 (1.17, 1.99)	−1.58 (−1.99, −1.17)	1.58 (1.17, 1.99)	<0.001
Fruit juices	0.24 (−0.06, 0.54)	−0.24 (−0.54, 0.06)	0.24 (−0.06, 0.54)	0.110
Saturated plant fats	−1.58 (−1.93, −1.23)	1.58 (1.23, 1.93)	−1.58 (−1.93, −1.23)	<0.001
Sugars and syrups	6.23 (5.84, 6.63)	−6.23 (−6.63, −5.84)	6.23 (5.84, 6.63)	<0.001
Misc. plant products	−0.62 (−0.92, −0.31)	0.62 (0.31, 0.92)	−0.62 (−0.92, −0.31)	<0.001
Animal food groups combined	0.39 (0.25, 0.53)			<0.001

Abbreviations: PDI, plant-based diet index; hPDI, healthful plant-based diet index; uPDI, unhealthful plant-based diet index; CI, Confidence Interval.

## Data Availability

Restrictions apply to the availability of these data. Data were obtained from Australian Bureau of Statistics and are available at https://www.abs.gov.au/about/data-services/consultancy-services#where-to-find-abs-data (accessed on 8 July 2024) with the permission of Australian Bureau of Statistics.

## Supplementary Materials

The following supporting information can be downloaded at: https://www.mdpi.com/article/10.3390/nu17071244/s1, Figure S1: Classification of plant-based diets applied to the calculation of PBD indices; Figure S2: Directed acyclic graph, representing the relations between variables of interest (PDIs and UPFs) and covariates; Table S1: STROBE-nut: An extension of the STROBE statement for nutritional epidemiology; Table S2: Plant-based diet indices (PDI, hPDI, uPDI) scoring system; Table S3: Component scores for PDI, hPDI and uPDI according to quintiles of UPF contribution in Australian adults from the 2011–2012 NNPAS; Table S4: Associations between the proportion of energy intake from UPFs and three diet quality indices (PDI, hPDI and uPDI) adjusted for sociodemographic variables (sex, age, education, country of birth, rurality, and area-level disadvantage) and energy misreporting (total energy intake to basal metabolic rate ratio) in Australian adults from NNPAS 2011–2012 according to age, sex and education (*n* = 9111).

## Abbreviations

The following abbreviations are used in this manuscript:

ACS	American Cancer Society diet score
AUSNUT	Australian Food and Nutrient Database
CI	confidence interval
FFQ	Food Frequency Questionnaire
hPDI	healthy Plant-based Diet Index
IQR	interquartile range
NNPAS	National Nutrition and Physical Activity Survey
PBD	plant-based diet
PDI	Plant-based Diet Index
Q	quintile
SD	standard deviation
SE	standard error
SEM	standard error measure
SSB	sugar-sweetened beverages
uPDI	unhealthy Plant-based Diet Index
UPFs	ultra-processed foods
UK	the United Kingdom
USA	the United States of America
