# Supplementary material for: Higher Consumption of Ultra-Processed Foods Is Associated with Lower Plant-Based Diet Quality in Australian Adults
Source: Nutrients. 2025 Apr 2;17(7):1244. doi: 10.3390/nu17071244 (PMC11990184; doi:10.3390/nu17071244)
Supplement: Supplementary file 1 [file nutrients-17-01244-s001.zip › nutrients-3552870-supplementary.pdf]

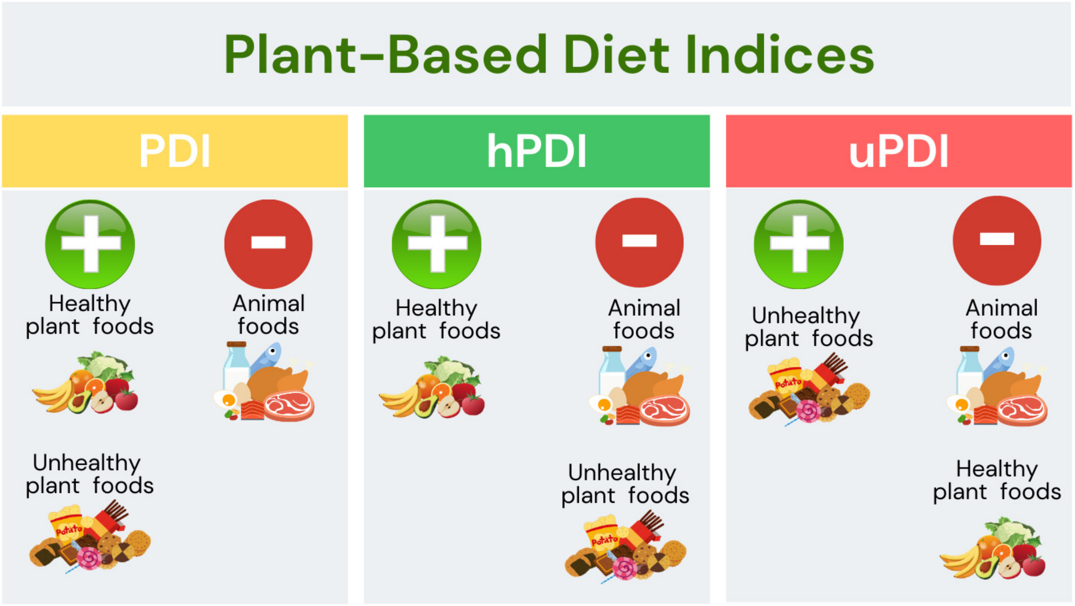

Supplementary Figure S1. Classification of plant-based diet applied for the calculation of PBD indices. Adapted from Satija et al. (38) PDI, plant-based diet index; hPDI, healthy plant-based diet index; uPDI, unhealthy plant-based diet index. (+) positive scoring, (-) reverse scoring.

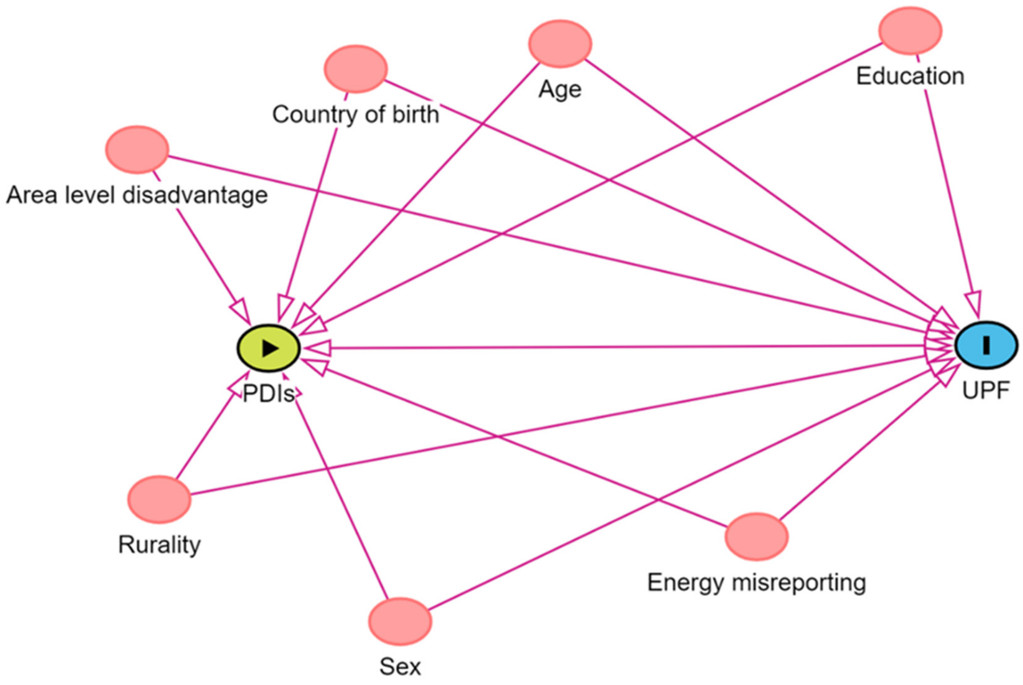

Supplementary Figure S2. Directed acyclic graph, representing the relations between variables of interest (PDIs and UPFs) and covariates.

Supplementary Table S1. STROBE-nut: An extension of the STROBE statement for nutritional epidemiology.

Item	Item Number	STROBE Recommendations	Extension for Nutritional Epidemiology Studies (STROBE-nut)	Page/Line
**Title and abstract**	1	(a) Indicate the study's design with a commonly used term in the title or the abstract	**nut-1.** State the dietary/nutritional assessment method(s) used in the title, abstract or keywords	p. 1-2
		(b) Provide in the abstract an informative and balanced summary of what was done and what was found		p. 1-2
**Introduction**				
**Background Rationale**	2	Explain the scientific background and rationale for the investigation being reported		p. 3-4
**Objectives**	3	State specific objectives, including any pre-specified hypotheses		p. 4<br>l. 69-71
**Methods**				
**Study design**	4	Present key elements of study design early in the study		p. 4-5
**Settings**	5	Describe the setting, locations and relevant dates, including periods of recruitment, exposure, follow-up and data collection	**nut-5.** Describe any characteristics of the study settings that might affect the dietary intake or nutritional status of the participants, if applicable	p. 4-5
**Participants**	6	(a) Cohort study – give the eligibility criteria and the sources and methods of selection of participants. Describe methods of follow-up	**nut-6.** Report particular dietary, physiological or nutritional characteristics that were considered when selecting the target population	NA
		Case–control study – give the eligibility criteria and the sources and methods of case ascertainment and control selection. Give the rationale for the choice of cases and controls		NA
		Cross-sectional study – give the eligibility criteria and the sources and methods of selection of participants		p. 4-5
		(b) Cohort study – for matched studies, give matching criteria and number of exposed and unexposed		NA
		Case–control study – for matched studies, give matching criteria and the number of controls per case		NA

**Variables**	7	Clearly define all outcomes, exposures, predictors, potential confounders and effect modifiers. Give diagnostic criteria, if applicable	**nut-7.1.** Clearly define foods, food groups, nutrients or other food components	p. 8-9
			**nut-7.2.** When using dietary patterns or indices, describe the methods to obtain them and their nutritional properties	p. 6-7
**Data sources – measurements**	8	For each variable of interest, give sources of data and details of methods of assessment (measurement). Describe comparability of assessment methods if there is more than one group	**nut-8.1.** Describe the dietary assessment method(s) (*e.g.* portion size estimation, number of days and items recorded), how it was developed and administered and how quality was assured. Report if and how supplement intake was assessed	p. 5
			**nut-8.2.** Describe and justify food composition data used. Explain the procedure to match food composition with consumption data. Describe the use of conversion factors, if applicable	p. 5
			**nut-8.3.** Describe the nutrient requirements, recommendations or dietary guidelines and the evaluation approach used to compare intake with the dietary reference values, if applicable	NA
			**nut-8.4.** When using nutritional biomarkers, additionally use the STROBE Extension for Molecular Epidemiology (STROBE-ME). Report the type of biomarkers used and their usefulness as dietary exposure markers	NA
			**nut-8.5.** Describe the assessment of non-dietary data (*e.g.* nutritional status and influencing factors) and timing of the assessment of these variables in relation to dietary assessment	NA
			**nut-8.6.** Report on the validity of the dietary or nutritional assessment methods and any internal or external validation used in the study, if applicable	p. 18<br>l. 367-370
**Bias**	9	Describe any efforts to address potential sources of bias	**nut-9.** Report how bias in dietary or nutritional assessment was addressed (*e.g.* misreporting, changes in habits as a result of being measured or	p. 5<br>l. 95-99<br>p. 9<br>l. 171-174

			data imputation from other sources)	
**Study size**	10	Explain how the study size was arrived at		p. 5<br>l. 85-91
**Quantitative variables**	11	Explain how quantitative variables were handled in the analyses. If applicable, describe which groupings were chosen and why	**nut-11.** Explain the categorisation of dietary/nutritional data (*e.g.* use of N-tiles and handling of non-consumers) and the choice of reference category, if applicable	p. 6-8
**Statistical methods**	12	(a) Describe all statistical methods, including those used to control for confounding. (b) Describe any methods used to examine subgroups and interactions. (c) Explain how missing data were addressed. (d) Cohort study – if applicable, explain how loss to follow-up was addressed. Case–control study – if applicable, explain how matching of cases and controls was addressed. Cross-sectional study – if applicable, describe analytical methods taking account of sampling strategy. (e) Describe any sensitivity analyses	**nut-12.1.** Describe any statistical method used to combine dietary or nutritional data, if applicable	p. 8-9
			**nut-12.2.** Describe and justify the method for energy adjustments, intake modelling and use of weighting factors, if applicable	p. 9<br>l. 183-186
			**nut-12.3.** Report any adjustments for measurement error (*i.e.* from a validity or calibration study)	NA
**Results**				
**Participants**	13	(a) Report the numbers of individuals at each stage of the study (*e.g.* numbers potentially eligible, examined for eligibility, confirmed eligible, included in the study, completing follow-up and analysed). (b) Give reasons for non-participation at each stage. (c) Consider use of a flow diagram	**nut-13.** Report the number of individuals excluded based on missing, incomplete or implausible dietary/nutritional data	p. 5
**Descriptive data**	14	(a) Give characteristics of study participants (*e.g.* demographic, clinical and social) and information on exposures and potential confounders. (b) Indicate the number of participants with missing data for each variable of interest. (c)	**nut-14.** Give the distribution of participant characteristics across the exposure variables if applicable. Specify if food consumption of total population or consumers only were used to obtain results	p. 10-11

		Cohort study – summarise follow-up time (*e.g.* average and total amount)		
**Outcome data**	15	Cohort study – report numbers of outcome events or summary measures over time. Case–control study – report numbers in each exposure category or summary measures of exposure. Cross-sectional study – report numbers of outcome events or summary measures		p. 11-13
**Main results**	16	(a) Give unadjusted estimates and, if applicable, confounder-adjusted estimates and their precision (*e.g.* 95% confidence interval). Make clear which confounders were adjusted for and why they were included. (b) Report category boundaries when continuous variables were categorised. (c) If relevant, consider translating estimates of relative risk into absolute risk for a meaningful time period	**nut-16.** Specify if nutrient intakes are reported with or without inclusion of dietary supplement intake, if applicable	p. 11-13
**Other analyses**	17	Report other analyses done (*e.g.* analyses of subgroups and interactions and sensitivity analyses.	**nut-17.** Report any sensitivity analysis (*e.g.* exclusion of misreporters or outliers) and data imputation, if applicable	NA
**Discussion**				
**Key results**	18	Summarise key results with reference to study objectives		p. 13
**Limitation**	19	Discuss limitations of the study, taking into account sources of potential bias or imprecision. Discuss both direction and magnitude of any potential bias	**nut-19.** Describe the main limitations of the data sources and assessment methods used and implications for the interpretation of the findings	p. 18
**Interpretation**	20	Give a cautious overall interpretation of results considering objectives, limitations, multiplicity of analyses, results from similar studies and other relevant evidence	**nut-20.** Report the nutritional relevance of the findings, given the complexity of diet or nutrition as an exposure	p. 13-17
**Generalisability**	21	Discuss the generalisability (external validity) of the study results		p. 17<br>l. 358-359
**Other information**				
**Funding**	22	Give the source of funding and the role of the funders for the present study and, if applicable, for the original study on which the present article is based		NA

**Ethics**		**nut-22.1.** Describe the procedure for consent and study approval from ethics committee(s)	p. 5<br>l. 83-84
**Supplementary material**		**nut-22.2.** Provide data collection tools and data as online material or explain how they can be accessed	See attachment

Supplementary Table S2. Plant-based diet indices (PDI, hPDI, uPDI) scoring system.

Group number	Food groups	Codes from plant-based diet database (47)	Food examples	PDI	hPDI	uPDI
Healthy plant food groups						
**1**	Whole grains	1000	Brown rice, barley, quinoa, whole oats, wholegrain or wholemeal wheat flour-based (bread, pasta)	P (+)	P (+)	R (-)
**2**	Fruits	1005	Apple, banana, other whole, fresh, frozen, canned or dried fruit	P (+)	P (+)	R (-)
**3**	Vegetables	1003	Broccoli, kale, peas, lettuce, potato, sweet potato, fresh and dried herbs and spices, 100% vegetable juice	P (+)	P (+)	R (-)
**4**	Nuts & seeds	1016	Nuts, seeds, nut butters, tahini, nut/seed pastes	P (+)	P (+)	R (-)
**5**	Legumes	1004	Beans, chickpeas, split peas, lentils	P (+)	P (+)	R (-)
**6**	Unsaturated plant oils & spreads	1017	Olive oil and other polyunsaturated and monounsaturated oils and margarine spreads	P (+)	P (+)	R (-)
**7**	Tea & Coffee	1018	Black tea, green tea, herbal tea, coffee	P (+)	P (+)	R (-)
Unhealthy plant food groups						
**8**	Refined grains	1002	White bread, white rice, couscous, cornflakes	P (+)	R (-)	P (+)
**9**	Fruit juices	1006	100% Fruit juices	P (+)	R (-)	P (+)
**10**	Saturated plant fats	1020	Coconut oil, palm oil, coconut cream/milk	P (+)	R (-)	P (+)
**11**	Sugars & syrups	1023	Sugar, agave syrup, maple syrup	P (+)	R (-)	P (+)
**12**	Misc. plant products	1021	Yeast and vegetable spreads, soy sauce	P (+)	R (-)	P (+)
Animal food groups						
**13**	Animal fat	1019	Butter, lard, ghee	R (-)	R (-)	R (-)
**14**	Low-fat dairy	1009	Reduced-fat or skim milk/yogurt/ricotta	R (-)	R (-)	R (-)
	Moderate-fat dairy	1008	Regular-fat milk, reduced-fat processed cheese	R (-)	R (-)	R (-)
	High-fat dairy	1007	Regular-fat cheese, halloumi	R (-)	R (-)	R (-)
**15**	Eggs	1015	Whole egg, egg yolks, egg whites	R (-)	R (-)	R (-)
**16**	Fish & Seafood	1014	Fresh and canned fish, prawns, scallops	R (-)	R (-)	R (-)
**17**	Red meats, lean	1010	Cooked lean red meats (fat<10%) such as beef, lamb, pork	R (-)	R (-)	R (-)
	Red meats, non-lean	1011	Cooked lean red meats (fat≥10%) such as beef, lamb, pork	R (-)	R (-)	R (-)
	Poultry, lean	1012	Cooked chicken, turkey, duck (fat<10%)	R (-)	R (-)	R (-)
	Poultry, non-lean	1013	Cooked chicken, turkey, duck (fat≥10%)	R (-)	R (-)	R (-)
**18**	Misc. animal-based foods	1022	Stocks (chicken, beef), Fish sauce, gravy, honey	R (-)	R (-)	R (-)

P (+) Positive score, R (-) Reverse score

Supplementary Table S3. Component scores for PDI, hPDI and uPDI according to quintiles of UPFs contribution in Australian adults from the 2011–2012 NNPAS.

**Food groups**	**PDI**					**hPDI**					**uPDI**				
	**Quintiles of the dietary contribution of ultra-processed foods (% of total dietary energy)***														
	**Q1**	**Q2**	**Q3**	**Q4**	**Q5**	**Q1**	**Q2**	**Q3**	**Q4**	**Q5**	**Q1**	**Q2**	**Q3**	**Q4**	**Q5**
Healthy plant food groups															
**Whole grains**	3.1 (0.05)	3.1 (0.04)	3.1 (0.05)	2.9 (0.05)	2.6 (0.05)	3.1 (0.05)	3.1 (0.04)	3.1 (0.05)	2.9 (0.05)	2.6 (0.05)	2.9 (0.05)	2.9 (0.04)	2.9 (0.05)	3.1 (0.05)	3.4 (0.05)
**Fruits**	3.2 (0.1)	3.3 (0.04)	3.0 (0.05)	2.9 (0.04)	2.5 (0.04)	3.2 (0.1)	3.3 (0.04)	3.0 (0.05)	2.9 (0.04)	2.5 (0.04)	2.8 (0.05)	2.7 (0.05)	3.0 (0.05)	3.1 (0.04)	3.6 (0.04)
**Vegetables**	3.3 (0.05)	3.2 (0.04)	3.0 (0.04)	2.9 (0.04)	2.6 (0.04)	3.3 (0.05)	3.2 (0.04)	3.0 (0.04)	2.9 (0.04)	2.6 (0.04)	2.7 (0.05)	2.8 (0.04)	3.0 (0.04)	3.1 (0.04)	3.4 (0.05)
**Nuts & seeds**	3.0 (0.05)	3.0 (0.05)	2.7 (0.05)	2.6 (0.05)	2.1 (0.05)	3.0 (0.05)	3.0 (0.05)	2.7 (0.05)	2.6 (0.05)	2.1 (0.05)	3.1 (0.06)	3.0 (0.05)	3.3 (0.05)	3.5 (0.05)	3.9 (0.05)
**Legumes**	1.9 (0.05)	1.7 (0.05)	1.8 (0.05)	1.7 (0.05)	1.5 (0.04)	1.9 (0.05)	1.7 (0.05)	1.8 (0.05)	1.7 (0.05)	1.5 (0.04)	4.2 (0.05)	4.3 (0.05)	4.2 (0.05)	4.3 (0.05)	4.5 (0.04)
**Unsaturated plant oils & spreads**	2.8 (0.04)	2.9 (0.04)	3.0 (0.05)	3.1 (0.04)	3.3 (0.05)	2.8 (0.04)	2.9 (0.04)	3.0 (0.05)	3.1 (0.04)	3.3 (0.05)	3.2 (0.04)	3.1 (0.04)	3.1 (0.05)	2.9 (0.04)	2.8 (0.05)
**Tea & Coffee**	3.2 (0.04)	3.2 (0.04)	3.1 (0.05)	2.9 (0.04)	2.6 (0.05)	3.2 (0.04)	3.2 (0.04)	3.1 (0.05)	2.9 (0.04)	2.6 (0.05)	2.9 (0.05)	2.8 (0.04)	2.9 (0.05)	3.1 (0.04)	3.4 (0.05)
Unhealthy plant food groups															
**Refined grains**	2.6 (0.05)	2.9 (0.04)	3.0 (0.05)	3.2 (0.04)	3.4 (0.04)	3.4 (0.05)	3.2 (0.04)	3.0 (0.05)	2.8 (0.04)	2.6 (0.04)	2.6 (0.05)	2.9 (0.04)	3.0 (0.05)	3.2 (0.04)	3.4 (0.04)
**Fruit juices**	2.1 (0.05)	2.5 (0.05)	2.7 (0.05)	2.9 (0.05)	2.7 (0.06)	3.9 (0.05)	3.5 (0.05)	3.3 (0.05)	3.1 (0.05)	3.3 (0.06)	2.1 (0.05)	2.5 (0.05)	2.7 (0.05)	2.9 (0.05)	2.7 (0.06)
**Saturated plant fats**	3.0 (0.05)	3.0 (0.05)	2.9 (0.05)	2.8 (0.05)	2.6 (0.05)	3.0 (0.05)	3.1 (0.05)	3.1 (0.05)	3.2 (0.05)	3.4 (0.05)	3.0 (0.05)	3.0 (0.05)	2.9 (0.05)	2.8 (0.05)	2.6 (0.05)
**Sugars & syrups**	2.0 (0.03)	2.7 (0.04)	3.1 (0.04)	3.5 (0.04)	3.8 (0.04)	4.0 (0.04)	3.3 (0.04)	2.9 (0.04)	2.6 (0.04)	2.2 (0.04)	2.0 (0.03)	2.7 (0.04)	3.1 (0.04)	3.5 (0.04)	3.8 (0.04)
**Misc. plant products**	2.6 (0.06)	2.7 (0.05)	2.6 (0.05)	2.5 (0.05)	2.6 (0.05)	3.4 (0.06)	3.3 (0.05)	3.4 (0.05)	3.5 (0.05)	3.8 (0.05)	2.6 (0.06)	2.7 (0.05)	2.6 (0.05)	2.5 (0.05)	2.6 (0.05)
Animal food groups combined	20.0 (0.1)	19.1 (0.1)	19.2 (0.1)	19.1 (0.1)	19.7 (0.1)	20.0 (0.1)	19.1 (0.1)	19.2 (0.1)	19.1 (0.1)	19.7 (0.1)	20.0 (0.1)	19.1 (0.1)	19.2 (0.1)	19.1 (0.1)	19.7 (0.1)

*Percentage of total energy intake from ultra-processed foods. Mean (range): Q1=11.2 (0-19.3), Q2=25.5 (19.3-31.4), Q3= 37.0 (31.4-42.9), Q4=50.2 (42.9-58.6), Q5=71.6 (58.6-100)

Supplementary Table S4. Associations between the proportion of energy intake from UPFs and three diet quality indices (PDI, hPDI and uPDI) adjusted for sociodemographic variables (sex, age, education, country of birth, rurality, and area-level disadvantage) and energy misreporting (total energy intake to basal metabolic rate ratio) in Australian adults from NNPAS 2011-12 according to age, sex and education (n=9111).

Subgroup		PDI		hPDI		uPDI	
		β (95% CI)	p	β (95% CI)	p	β (95% CI)	p
Age (years)	19-30	-0.4 (-0.65, -0.15)	0.002	-0.76 (-0.97, -0.54)	<0.001	0.86 (0.64, 1.07)	<0.001
	31-50	-0.18 (-0.31, -0.04)	0.012	-0.71 (-0.82, -0.60)	<0.001	0.80 (0.67, 0.93)	<0.001
	51-70	0.09 (-0.06, 0.24)	0.249*	-0.51 (-0.66, -0.36)	<0.001	0.65 (0.51, 0.80)	<0.001
	71+	0.02 (-0.22, 0.25)	0.89*	-0.52 (-0.72, -0.33)	<0.001	0.98 (0.79, 1.17)	<0.001
Sex	Male	-0.15 (-0.27, -0.02)	0.03	-0.63 (-0.74, -0.52)	<0.001	0.69 (0.57, 0.82)	<0.001
	Female	-0.11 (-0.24, 0.02)	0.1	-0.67 (-0.78, -0.56)	<0.001	0.93 (0.81, 1.04)	<0.001*
Education	Low	0.02 (-0.17, 0.20)	0.9	-0.55 (-0.70, -0.41)	<0.001	0.73 (0.58, 0.88)	<0.001
	Medium	-0.12 (-0.25, 0.02)	0.09	-0.65 (-0.77, -0.52)	<0.001	0.76 (0.64, 0.88)	<0.001
	High	-0.28 (-0.44, -0.12)	0.001*	-0.74 (-0.87, -0.60)	<0.001	0.93 (0.76, 1.10)	<0.001*

Abbreviations: PDI, plant-based diet index hPDI, healthful plant-based diet index, uPDI, unhealthful plant-based diet index; CI, Confidence Interval;

Values are regression coefficients (95% CIs) estimated with multiple linear regressions. *p-value for interaction <0.05.*
